# Smart Mobility and Smart Climate: An Illustrative Case in Seville, Spain

**DOI:** 10.3390/ijerph20021404

**Published:** 2023-01-12

**Authors:** María Eugenia López-Pérez, María Eugenia Reyes-García, María Eugenia López-Sanz

**Affiliations:** Área Departamental Ciencias Sociales y de la Salud, Centro Universitario San Isidoro, 41092 Sevilla, Spain

**Keywords:** sustainability, smart mobility, climate change, ODS, case study, Spain

## Abstract

In recent years, smart city projects and initiatives have surged around the globe. Yet, a wide range of factors determine the success or failure of such initiatives and there is still a long road ahead in terms of effective governance and innovation management. In such a context, this study explores the specific case of PCT Cartuja (science and technology park in Seville, Spain)—analyzing several smart-mobility and smart-climate solutions from a Triple Helix Model standpoint. The authors tap into multiple information sources to describe the case and key implications of smart initiatives for both theory and management are discussed. This paper shows the current progress as well as the remaining challenges to illustrate how public-private partnerships and conflict can be effectively managed.

## 1. Introduction

At a time when an estimated 70% of the world’s population live in urban areas, the pressure exerted on certain ecosystems—coupled with unprecedented climate change and migratory movements—continue, in many cases, to fuel makeshift urbanization processes, ratcheting up the intensity and impact of urban crises [1]. The United Nations [2] recognizes that rapid urbanization processes are increasingly triggering serious problems: air pollution, traffic congestion, slums and marginal areas, and unbalanced infrastructure provision are just a few examples of urban planning and management malpractice.

A number of cities, however, are becoming drivers of sustainable development, equality, inclusion, cultural diversity and innovation [3]. There is also evidence of cities which, following an analysis and strategic planning process, have managed to reverse the impact of negative externalities linked to urban overpopulation—becoming models of effective urban planning practices; among them are Medellín, Colombia [4], Milan, Italy [5] and Shanghai, China [6]. Others, including Delhi, India, have achieved this via practices aimed at enhancing mobility in a number of metropolitan areas [7]. In such cases, smart-city solutions are especially attractive [8]. Moreover, Information and Communication Technologies (ICTs) are increasingly widespread—allowing existing and new infrastructures to be effectively leveraged to (re)design urban planning and management. ICTs are also useful in city services provision. Most developed and a handful of developing countries are implementing smart city solutions to address urbanization-related problems [9,10,11].

In this vein, urban planners and managers are contributing to the following Sustainable Development Goals (SDGs): health and well-being (SDG 3), affordable, clean energy (SDG 7), sustainable cities and communities (SDG 11), climate action (SDG 14) and life of terrestrial ecosystems (SDG 15), among others.

The literature on smart cities is both abundant and broad; yet, as authors such as Medina-Molina et al. [12] suggest, more cases addressing the dynamics of real processes contributing to real change would be of interest. It would then be possible to identify interactions between agents, resources and contexts involving some type of innovation, be it radical or incremental—or simply to implement ideas adapted from successful experiences with a view to better understand smart (re)urbanization processes which contribute to improving citizen quality of life.

To this end, the present study examines a real-life case, currently under development in pilot plan phase: the smart PCT Cartuja project in Seville, Spain. The authors illustrate smart-mobility and smart-climate processes, within the framework of a global smart district plan and taking the Triple Helix Model [13] as our reference. To this end, the second section presents the smart city concept from a dynamic perspective and shows the Triple Helix Model to be the ideal frame of reference for analyzing the phenomenon under study. The third section includes our methodological approach, and illustrative and qualitative case study and pertinent information regarding the smart PCT Cartuja project case. Finally, a theoretical discussion and practical implications are presented, based on our most relevant findings.

## 2. Literature Review: Smart Cities as an Evolving Concept

The definition of “smart city” is not static; the concept is dynamic, evolving along with conceptions of urban vision and planning. As Cambra-Fierro and Pérez [14] and Oliveira et al. [15] indicate, the smart city literature dates back to the year 1990, with the term originally referring to cities harnessing new technologies to streamline infrastructure design and management. Around the year 2000 the focus shifted. Key to the new smart city conception was the use of information technologies (ITs), giving rise to the idea of “ubiquitous city” or U-city [10], in reference to the ability to solve urban problems any time, any place using IT [16]. In many cases, however, this remained a mere declaration of intent, as implementing ITs in urban planning was no easy task. Hence, in the period 2006–2010, which has been called the “IT infrastructure construction stage” gains prominence [10]; while the U-city concept only leveraged IT to solve general urban problems, the smart city concept started matching IT to the specific problems of citizens themselves [10,17,18]. Thus, “smart” begins to be understood in terms of citizen-oriented services—based on data collected regarding the most relevant aspects of city operation, including transport, administration, risk and disaster prevention, the environment and well-being, among others. The more authorities invest in infrastructure, the more IT’s capacity to mitigate or solve urban problems is perceived. Yet, as might be expected, implementation is far from automatic. Hence, authors such as Kim [10] and Leem et al. [19] speak of an essential early stage for smart city services to get up and running. Specifically, *“…to introduce Smart city services, urban problems need to be defined before they can be solved…and Smart city services must be introduced on a trial basis in designated areas”* [10] (p. 2). At this stage, objectives center more around operational aspects than on actual performance. As more projects are implemented, however, more realistic estimates regarding smartization, urban planning and city service capacities can be obtained—allowing urban challenges to be defined in advance [20]. Hence, plans can be made for building smart cities and developing smart solutions aimed at solving urban problems—implying smart technologies are capable of both delivering practical services tailored to residents’ needs and proactively anticipating and meeting urban challenges [21,22]. As the smart-city concept matures and smart approaches are increasingly capable of responding to emerging threats and demands, efficiency is maximized through provision of flexible smart services [21].

Throughout this temporal process, the evolution of the concept suggests that smart city approaches must go beyond a mere ICT-centered focus to adapt to the needs and expectations of individual citizens and the larger community as a whole [14]. A real concern for quality of life should also be present—both in terms of the opportunities afforded and economic, social and environmental development [23]. In sum, the interplay between technology, the economy, society and the environment is key to guaranteeing the well-being of citizens and success of the smartization process [24].

The literature (e.g., [14,25,26,27,28,29,30]) identifies a series of elements, essential to fully understanding the smart city concept at all levels: infrastructure represents the base, starting with an sufficient supply of electricity and communication perspective (i.e., IT infrastructure and social overhead capital). Data, continuously generated within the smart infrastructure, is another essential ingredient. In particular, attention must be paid to collecting, storing, processing and analyzing this data—here, approaches such as big data and artificial intelligence are very useful. Thirdly, citizens using smart devices connected to the smart city infrastructure and willing to share their data. Finally, very clear objectives with regard to city smartization—from a global smart city vision to resolution of specific urban challenges such as traffic jams, disaster prevention/management, environmental pollution and garbage collection, etc.—ensuring a satisfactory level of quality of life [14].

The smart-city outlook is to provide a fresh, citizen-centered vision of urban environments; hence, recognizing the essential role citizens play [15]. The overarching goal of any smart approach, then, should be to facilitate urban planning, construction and management with special attention paid to providing citizens with smart services—the aim being to guarantee the best possible quality of life. In so doing, the primary focus should be to leverage ICTs and other smart tools and devices to better integrate citizens into the urban environment, fostering more participatory decision-making processes and increased transparency [15].

**Figure 1:**
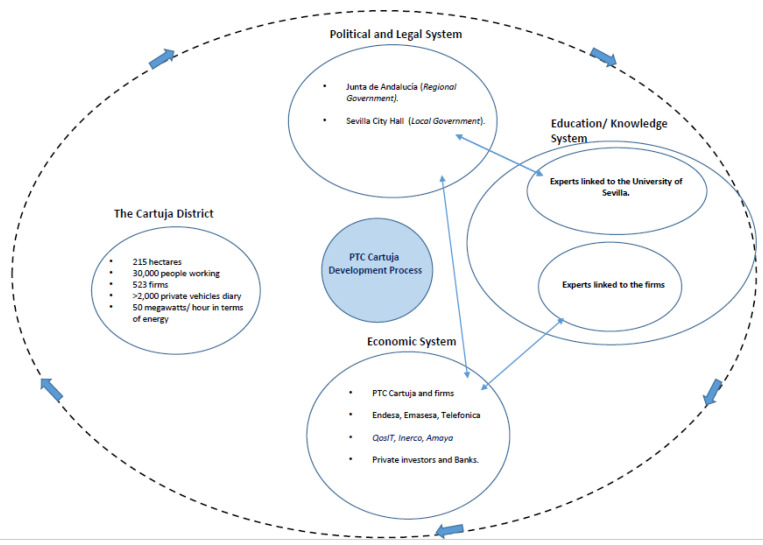
Outline of the PTC Cartuja project using the Triple Helix Innovation framework. Source: Adapted from Etzkowitz and Leydesdorff (1995).

In this context, the Triple Helix Model, proposed by Etzkowitz and Leydesdorff [13], serves as a model of collaborative governance rooted in joint public-private/citizen decision-making. The model considers relationships between three main elements: (i) *the education system*, representing human capital (e.g., universities and research centers), (ii) *the economic system* (economic capital: industry, service firms, banks), and (iii) *the political system* (political and legal capital: national, regional and local government institutions). It allows for understanding collaborative governance based on joint public-private/citizen decision-making in a smart and sustainable urban planning process. Figure 1 shows the outline of the PTC Cartuja project using the Triple Helix Innovation framework.

## 3. Method and Data

Our methodology of choice is an illustrative qualitative case study. This approach is commonly accepted in urban research [4,23]. The unit of analysis is the PCT Cartuja Project. Case choice is based on the authors’ assessment of the smart PCT Cartuja project in Seville, Spain as being a very innovative, sustainable project. Our study’s data derive from secondary sources and in-depth interviews with key informants. We developed five semi-structured interviews (CEO, PCT Cartuja; Mobility Delegate, Seville City Council; Director of Institutional Relations, Endesa Energy; Technical Supervisor, Emasesa Water; and CEO, QosIT Consulting) representing the three dimensions of the Triple Helix Model. Interviews averaged between 30 and 40 min. Semi-structured interviews presented an introductory phase aiming to clarify the objectives of the research and providing a framework to keep the discussion on the topic, while allowing the flexibility to identify new issues [14,31]. Data from interviews were complemented with additional evidence from press articles, web pages and visits to the PTC Cartuja District. These supplementary sources helped to match information derived from the interviews. All of the interviews were transcribed. Moreover, the interview notes were sent to each of the participants so that they could include additional data and/or confirm the preliminary results, as literature suggests [31,32,33].

Two main pathways related with the project emerged: smart mobility and smart climate issues. In terms of smart mobility, data highlighted subtopics related with increasing the number of pedestrian areas, the use of apps, e-vehicles and shared mobility; while in terms of smart climate, data reveal interests in the use of renewable energy and temperature control. In what follows, we present the main data related with the PCT project, as well as the interplay between the Triple Helix Model dimensions and the topics/sub-topics identified in the data analysis.

### 3.1. PCT Cartuja, Seville (Spain)

In the early 1990s, Seville’s Isla de la Cartuja district experienced an initial phase of large-scale urban transformation, in preparation for Expo ’92. After the event, some buildings were dismantled while swaths of the Expo grounds and infrastructure fell into disuse and relative abandonment; others were repurposed to house a university campus, public institutions and firms—giving birth to the science and technology park district known today as PCT Cartuja. More recently, a range of urban regeneration and redesign projects aimed at revitalizing the district have led to the incorporation of green spaces, a shopping area and the 40-story Torre Sevilla skyscraper housing a hotel, a panoramic restaurant and office space.

**Figure 2:**
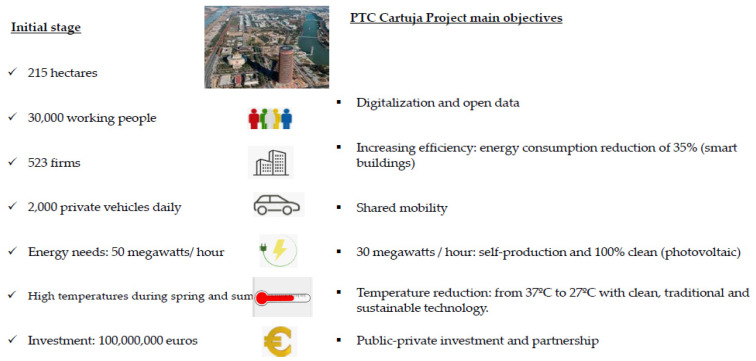
PTC project main figures. Source: https://www.pctcartuja.es/, (accessed on 3 January 2023), press articles and interviews.

PCT Cartuja is a unique urban area, just a 10-min walk from Seville’s city center, lacking residential buildings, and bordered by a river, on one side and a dock area on the other—making for a suitable experimental environment. Receiving over 30,000 people daily and generating 2% of Andalusia’s GDP, PCT Cartuja is a laboratory for the city of the future—aspiring to reach clean energy self-sufficiency, partially restricted to traffic and boasting sustainable, autonomous distribution systems. The district currently serves as test bench for an innovative eco-friendly outdoor air conditioning system inspired by 2000-year-old hydro-geological technology from the Near East, capable of lowering ambient temperatures by 10 degrees. Moreover, there is an environment of full cooperation between political authorities at the local, regional and national level in the smart PCT Cartuja project, as well as the active participation of a number of private companies who view the project as a unique laboratory opportunity. Hence, the smart PCT Cartuja project presents opportunities on several fronts, including smart mobility and smart climate. Figure 2 shows the project main figures in terms of the starting point and main objectives.

*“Initially, the challenge was to transform Isla de la Cartuja into the host site for Expo ’92…later, to convert all Expo assets into a space for innovation and technology, aimed at helping transform part of the Andalusian economy along three axes: university, business and science & technology. Now we are running out of space and there are new buildings planned on the few available plots… Seeing that the ecosystem works, we have to think about where we want to go. Three years ago, we began to design the transformation of PCT Cartuja into the smart city of the future, with a public-private investment of around 100 million euros.”*

(CEO, PCT Cartuja)

### 3.2. Smart Mobility

PCT Cartuja accommodates a conglomerate of business and institutional buildings, accessible via several urban bus lines and a system of bike lanes. It boasts large areas for free private-vehicle parking and two private pay-for-time parking lots—almost all vehicles, however, are concentrated in the vicinity of the main buildings. Moreover, most workers’ schedules are similar, especially with regard to morning entry and exit times—often triggering traffic jams at the beginning and end of the workday due to badly parked vehicles. Evenings, weekends and holidays, the pressure on vehicle space drops off very sharply or ceases to exist all together.

In this context, the project calls, for instance, for the semi-pedestrianization of a number of streets, combining more green areas. There are also plans to reorder traffic and current parking spaces within the district and surrounding areas using an eco-friendly permit system requiring access via a smart parking application and camera ID. Ride planning is also considered with solar pergolas to power a system of electric recharge points and more bike-parking slots are expected to promote the use of bicycles. Other alternatives such as e-scooters and public transport electrification are being discussed.

*“We have designed and activated a smart mobility app…MathIT.MOTUS…created to monitor the movement of all people working in the Cartuja area […]. We can monitor user journeys from their homes, commute schedules, means of transport […] However, people must become active agents…we need real, accurate information to design efficient models and attempt to boost well-being and overall satisfaction.”*

(CEO, QosIT Consulting)

In terms of mobility, authorities and planners stimulate sustainable models, highlighting more space for pedestrians and cyclists, e-vehicle use via a new network of recharging stations and other infrastructure. The basic assumption is that the future of mobility will be electric and shared; the objective is substituting by 2025 of roughly 2000 carbon-emission vehicles with e-vehicles, with a special consideration for priority access to the district for clean mobility users.

*“We have a global objective with an initial milestone: reaching up to 20% of the total share of electric vehicles in 2025 and 37% in 2030… Installation of at least 200 recharging points within PCT Cartuja is also planned.”*

(Mobility Delegate, Seville City Council)

*“Starting in 2023 we will have two low-emission areas where gasoline vehicles registered prior to the year 2000 and diesel vehicles registered before 2006 will not be allowed to circulate. To this, we add reinforcement of the public transport system, shuttles and bike lanes…which, it is true, will have to make up for the lack of a metro line and the truncated commuter train landing.”*

(Antonio Muñoz, Mayor of Seville)

### 3.3. Smart Climate

In the environmental and energy field, public-private collaboration is clearly present. The electric utility company, Endesa, for instance, will invest 30 million to ensure all energy consumed within PCT Cartuja comes from local renewable sources.

*“PCT Cartuja needs 50 megawatts per hour and year… for this we are going to invest the necessary resources and invite other well-established companies with self-generation and efficient building formulas, with the support of the Andalusian Regional Government and leadership of the Energy Agency, to reduce consumption for all PCT Cartuja buildings by at least 35%. And, of course, any new buildings must comply with specific sustainability and efficiency characteristics.”*

(CEO, PCT Cartuja project)

*“We want PCT Cartuja to be a clear, lively showcase, where resident companies can teach the technology to other clients…a laboratory, where what is achieved in Cartuja can be perfectly applicable to the rest of the city of Seville and to any other part of the world.”*

(Antonio Muñoz, Mayor of Seville)

*“The entire system will be monitored through a large digital technology platform that will provide solidarity-centered energy management, delivering any surplus to needy infrastructures and to mobility.”*

(Director of Institutional Relations, Endesa Energy)

However, from an environmental standpoint, another great challenge arises: reducing the ambient temperature—especially during the summer months, when temperatures easily exceed 40 degrees Celsius. A project has been launched to lower the ambient temperature from 37 to 27 degrees Celsius (currently, the technology is not effective in temperatures of 40 degrees Celsius and above).

*“To meet this challenge we are designing an outdoor air conditioning system…reusing bioclimate systems from Expo ‘92 and readapting them. This new plan is called Cartuja Qanat and is financed by the EU Urban Innovation Action program (80%) and promoted by the local water management company, Emasesa, the Urban Management and Employment departments at Seville City Hall, the CSIC, University of Seville, PCT Cartuja and Innovarcilla. This partnership has already invested 5 million euros in the project, of which 3.5 million have been allocated to infrastructure projects.”*

(CEO, PCT Cartuja)

*“The system includes water tanks and canals (qanat), air-cooling tubes and covered, semi-buried spaces designed to resist heating from the sun. Simply explained, the tanks store water which, once cooled, flows into the canals. Here, warm air from the exterior flowing through the tubes cools in contact with the cooled water and is injected into the covered spaces at a much lower temperature.”*

(Technical Supervisor, Emasesa Water)

Water cooling is maximized by pulverizing the water throughout the night. In addition, the system’s protective cover incorporates photovoltaic panels—supplying solar energy during the day and, at night, providing a flat surface onto which water is poured in a very thin sheet to cool via radiation. Significantly, the system’s architectural design considers aspects such as orientation towards prevailing winds for air intake and thermal issues involving use of water microdiffusers. The entire system is 100% self-sufficient, powered by energy generated onsite.

*“It’s basically the way an air conditioner works, but the cooling method is natural. There is an additional temperature control system, similar to a radiator or underfloor heating, but instead of being located underfoot it is located on the ceiling. The important thing about this project is that it is a test experience aspiring to be replicated throughout the city, helping combat rising temperatures due to climate change…in short, allowing citizens to enjoy city streets again with solutions adapted to each specific space.”*

(Technical Supervisor, Emasesa Water Co.)

More often than would be desirable, contemporary urban planners have eliminated green areas in favor of hard, practical, artificial spaces; they are more economical and easier to maintain, yet with harmful repercussions for the environment and the climate. Hence, in an effort to adapt to global warming, the experimental Cartuja Qanat natural cooling system is being considered for replication in other urban areas, including bus stops, pedestrian crossings, squares and schoolyards—not only in Seville but across Andalusia via the EU-funded Life Watercool project.

*“The threat of global warming has intensified and with this project we can combat the multiple consequences of climate change, but not the causes. Southern European cities are especially vulnerable to the impact of climate warming and many spaces designed for citizens’ enjoyment, such as playgrounds, receive intense solar radiation…in practice, preventing their use. We want to reverse this situation.”*

(Principal Researcher, Cartuja Qanat Project—University of Seville)

*“One must be very careful when intervening in cities. Road traffic creates many problems, not just traffic jams…we must create car-free and combustion-free zones and rethink cities so that public spaces make more sense. Urban vegetation is a fundamental factor in bioclimatic conditioning…clean energy sources are as well.”*

(Principal Researcher, Cartuja Qanat Project—University of Seville)

## 4. Discussion and Conclusions

The case of PCT Cartuja reveals very interesting insights in terms of sustainability and quality of life for citizens. Based on the Triple Helix Model scheme, data identify issues related with smart mobility (i.e., pedestrian areas, e-vehicles, shared mobility and the use of apps for mobility management) and smart climate (i.e., use of renewable energy and temperature control) which could be extrapolated to other contexts.

At a time when humanity is facing unprecedented challenges such as climate change, extreme food and resource shortages, soaring global population and spiking rural emigration flows—intensifying demographic pressure in urban areas—smart city-based solutions for urban planning and management seem, more than an option, a necessity. While the smart city literature proposes a range of definitions, on the whole, use of ICTs to improve municipal services and infrastructures, benefit the environment, streamline resource management and enhance citizen well-being can be assumed [9]. Naturally, most smart city initiatives entail service and product innovations [34] and city managers must not neglect their duty to develop and deliver services to citizens (e.g., housing, mobility, telecommunications, transportation, lighting and leisure, etc.). Moreover, urban energy infrastructure, public and private transport, buildings and green spaces must be properly designed and managed in terms of sustainability, as Razmjoo et al. [8] have indicated recently (clean energy and CO_2_-emission reduction have become key elements, for instance). Smart cities, then, can be seen as a new urban development paradigm that can increase service efficiency and quality of life, while minimizing the environmental impact, as authors such as Bibri [35] have pointed out. In this context, illustrative success stories are a good medium for analyzing transition towards smart-city designs—as well as for identifying effective policies throughout such processes.

As Oliveira et al. [15] (p. 7) suggest, “emerging technologies and Smart city models/paradigms are bringing a new wave of decentralized approaches both in terms of resource allocations, governance models and opportunities for innovation”; in the case at hand, the smart PCT Cartuja project in Seville (Spain), it is evident and governance is indeed a fundamental pillar requiring further reflection [36]. The city (i.e., city managers) assumes a broad spectrum of roles, from services provider to regulator, enabler and services consumer [37]. Likewise, there is no doubt regarding the importance of coordination among different levels of government (i.e., local, regional, national) and engagement with other stakeholders (e.g., citizens, private firms). PCT Cartuja boasts an unusual degree of coordination at all levels of government, regardless of representing rival political parties. Rare “across-the-aisle” cooperation of this sort may be encouraged or required by the fact that the pilot plan involves large buildings belonging to different government agencies, availability of considerable EU funding and/or the large number of private companies which have shown an interest in being involved; whatever the case, this environment encourages public-private collaboration insofar as it reduces uncertainty and injects stability into the project. On the whole, it appears decisions made by both city and regional governments support stakeholder interests; administrators, on the other hand, can easily access stakeholder knowledge, as a number of stakeholders are experts in their fields or users of smart features and services linked to the PCT Cartuja project, in line with Razmjoo et al. [8]. More specifically, we note for instance that all stakeholders support investment in sustainability and renewable energy, e.g., prioritizing sustainable/public transport solutions over conventional private vehicle options—even when this requires investing in charging stations or new bus lines. Clearly, interdependencies arise and must be managed effectively.

Secondly, technology and innovation undoubtedly play a fundamental role in defining how the smart city concept is implemented (e.g., [38]). Smart cities recognize the crucial role of ICTs in terms of boosting operational efficiency, enhancing service quality, promoting sustainable practices and guaranteeing citizen well-being [39]—hence, technology development is essential to making this possible. Increasing efficiency can make technology more productive, eco-friendly and agile; such advances also empower better resource management. In the case of the smart PCT Cartuja project, a key line of action is the city-energy-sustainability link, where clean, renewable energy production is fundamental [40,41]. Clear examples of this are the Cartuja Qanat subproject—aimed at developing a sustainable climate ecosystem to deliver milder temperatures; energy company Endesa’s commitment to experimenting with clean energy generation and storage processes; and an algorithm—requiring active user participation—designed to streamline and enhance the transport system and ease traffic.

From a practical standpoint, we observe the presence and interrelation of a wide range of stakeholders in the smart PCT Cartuja project. Hence, we have proposed the Triple Helix Model [13] as our reference—a model of collaborative governance based on public-private/citizen joint decision-making. With regards to governance, we noted earlier the importance of the political helix; at all levels, project responsibilities, priorities and plans have been established in a coordinated manner; from an organizational and general administration standpoint, reducing uncertainty and ensuring project stability and successful development. The economic helix is also apparent, as private capital firms and public institutions coexist and collaborate. For all agents involved, the smart PCT Cartuja project represents an ideal test bench, insofar as tested solutions can later be extrapolated to other contexts—two clear examples being Endesa’s innovative clean energy generation, distribution and storage systems and QosIT Consulting’s MathIT smart sustainable mobility platform. Moreover, in this innovative context, the education system helix is also decisive—e.g., the proposed Cartuja Qanat solution aimed at reducing ambient temperatures springs from a collaborative University of Seville-led project, with potential for implementation in a number of smart urban planning/development contexts beyond PCT Cartuja. Likewise, the proposed MathIT smart mobility solution springs from a private firm research, development and innovation (I + D + i) project aimed at cutting back private vehicle traffic in the area and enhancing user well-being by reducing traffic jams and pollution.

Hence, we observe that very satisfactory results can be expected when all three dimensions of the Triple Helix Model interact effectively. Moreover, the primary mission of any urban design actions aimed at true smart-city transformation must be to have a real, positive social, human and environmental impact [39]. Moreover, while authors such as Kim [10] identify varying degrees of development with regard to the smart city concept, pilot projects remain a necessity. Finally, access to contexts such as the smart PCT Cartuja project—boasting both private investment and public funding and serving as a real-life laboratory for observing governance dynamics and testing solution effectiveness, efficiency, robustness and scalability in an environment of innovation and interdependence—is a priceless opportunity for many stakeholders.

From a management perspective, with a view to generate positive dynamics, this case allows us to identify the following relevant factors:

- Existence of a clear, transparent governance mechanism—where all agents know exactly what level of involvement is expected, which resources must be invested and the individual/global output that can be expected;
- Explicit commitment of all stakeholders, regardless of being public or private.
- Clear, fluid mechanisms for dialogue and coordination among managers, companies and users, insofar as interrelationships among all exist;
- Planning and prioritization of objectives within an open dialogue framework which, on occasions, can lead to change management scenarios.

Despite the relevance of this research, it is true that our discussion and conclusions are based on analysis of a single case. This is an ongoing project with a positive dynamic, defined by an apparently adequate structure of governance. That said, the smart PCT Cartuja project represents an illustrative success case and—as authors such as Medina-Molina et al. [12] suggest—more cases shedding light on the dynamics of real-life situations, contributing to real change and better implementation of smart solutions (in our case linked to mobility and climate), are always of interest.

Our proposal for future research is to delve deeper into the ideas presented here—analyzing other success-failure cases, perhaps in different socio-economic and/or cultural contexts [9] or considering a different set of smart solutions [39]. Issues related with public-private partnerships, potential conflict management and co-creative process would be of interest in terms of governance. Identifying specific initiatives related with smart-mobility and climate change, as well as their potential to be extrapolated to other contexts would be relevant in terms of innovation management. However, all of the initiatives should be considered in the broad perspective of urban planning and management with references to sustainability and the citizens’ quality of life.

## Data Availability

Not applicable.
